# Supplementary material for: Optimization of Bilayer Resistive Random Access Memory Based on Ti/HfO2/ZrO2/Pt
Source: Materials (Basel). 2024 Apr 17;17(8):1852. doi: 10.3390/ma17081852 (PMC11051404; doi:10.3390/ma17081852)
Supplement: Supplementary file 1 [file materials-17-01852-s001.zip › materials-2919516-supplementary.pdf]

materials

Supporting information

Optimization of Bilayer Resistive Random Access Memory based on Ti/HfO_2/ZrO_2/Pt

Zhendong Sun [1], Pengfei Wang [2], Xuemei Li [1], Lijia Chen [1], Ying Yang [1] and Chunxia Wang [1,*]

[1] College of Physics and Electronic Engineering, Chongqing Normal University, Chongqing 401331, China; mgzdsun01@163.com (Z.S.); 18716218339@163.com (X.L.); ljchen01@cqnu.edu.cn (L.C.); 20131184@cqnu.edu.cn (Y.Y.)

[2] Analog Foundries Co., Ltd., Chongqing 401332, China; pndn7712@163.com

* Correspondence: 20132078@cqnu.edu.cn

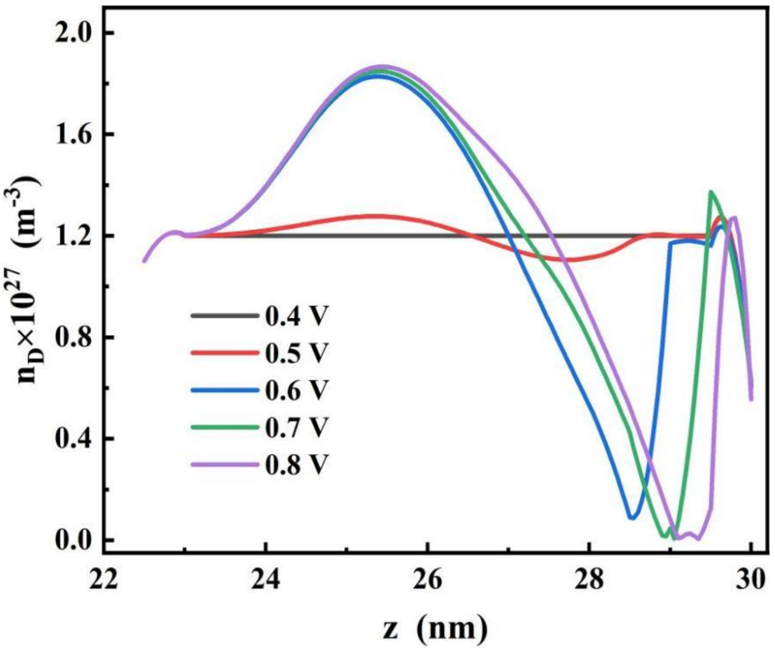

Figure S1. One−dimensional distribution curves of oxygen vacancy concentration inside CF1 and CF2 of Ti/HfO_2/ZrO_2/Pt at bias voltages 0.4 V−0.8 V (step by 0.1 V) in the SET process.

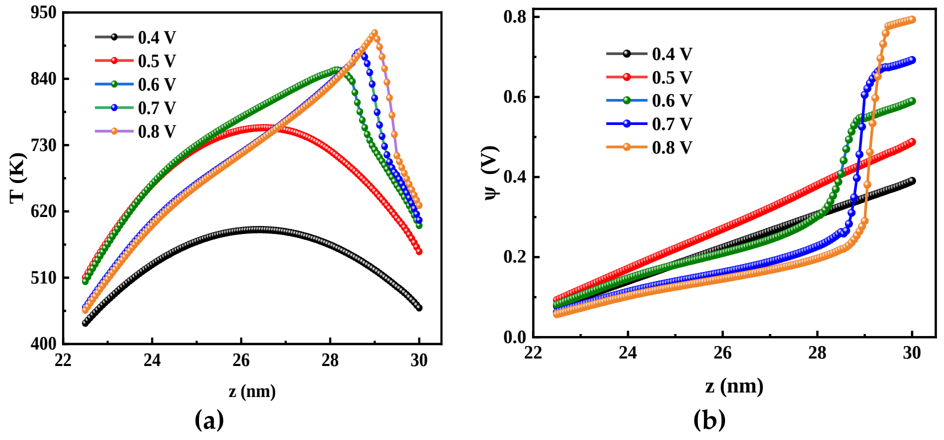

Figure S2. One−dimensional distribution curves in the SET process of Ti/HfO_2/ZrO_2/Pt at bias voltages 0.4 V- 0.8 V (step by 0.1 V): **(a)** temperature (T); **(b)** electric potential (Ψ).

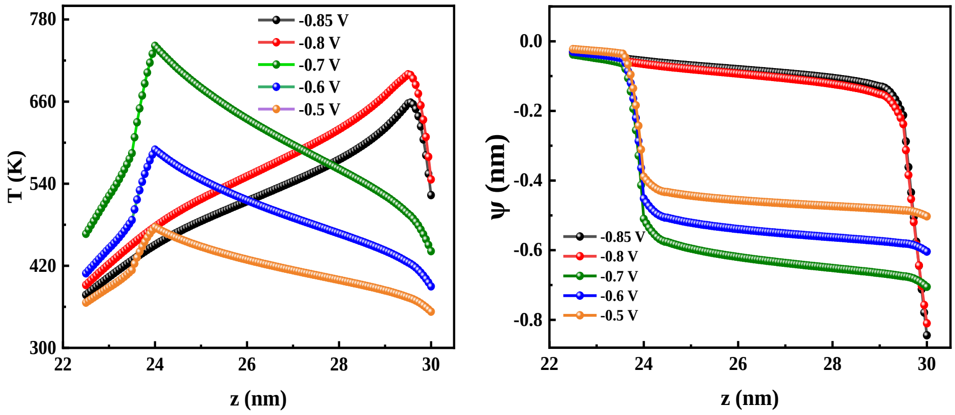

(a) **(b)**

Figure S3. One−dimensional distribution curves in the SET process of Ti/HfO_2/ZrO_2/Pt at bias voltages -0.85V, -0.8V, -0.7V, -0.6V, and -0.5V: **(a)** temperature (T); **(b)** electric potential (Ψ)
